# Toward a neuroaesthetics of interactions: Insights from dance on the aesthetics of individual and interacting bodies

**DOI:** 10.1016/j.isci.2025.112365

**Published:** 2025-04-08

**Authors:** Andrea Orlandi, Matteo Candidi

**Affiliations:** 1 Department of Psychology, Sapienza University, Rome, Italy; 2 IRCCS Santa Lucia Foundation, Rome, Italy; 3 School of Psychological Sciences, Macquarie University, Sydney, NSW, Australia

**Keywords:** Neuroscience, Cognitive neuroscience, Social sciences

## Abstract

Neuroaesthetics has advanced our understanding of the neural processes underlying human aesthetic evaluation of crafted and natural entities, including the human body. While much research has examined the neurocognitive mechanisms behind evaluating “single-body” forms and movements, the perception and aesthetic evaluation of multiple individuals moving together have only recently gained attention. This review examines the neural foundations of static and dynamic body perception and how neural representations of observed and executed movements influence their aesthetic evaluation. Focusing on dance, it describes the role of stimulus features and individual characteristics in movement aesthetics. We review neural systems supporting visual processing of social interactions and propose a role for these systems in the aesthetic evaluation of interpersonal interactions, defined as the neuroaesthetics of interactions. Our goal is to highlight the benefits of integrating insights and methods from social cognition, neuroscience, and neuroaesthetics to understand mechanisms underlying interaction aesthetics, while addressing future challenges.

## Graphical abstract

**Figure:**
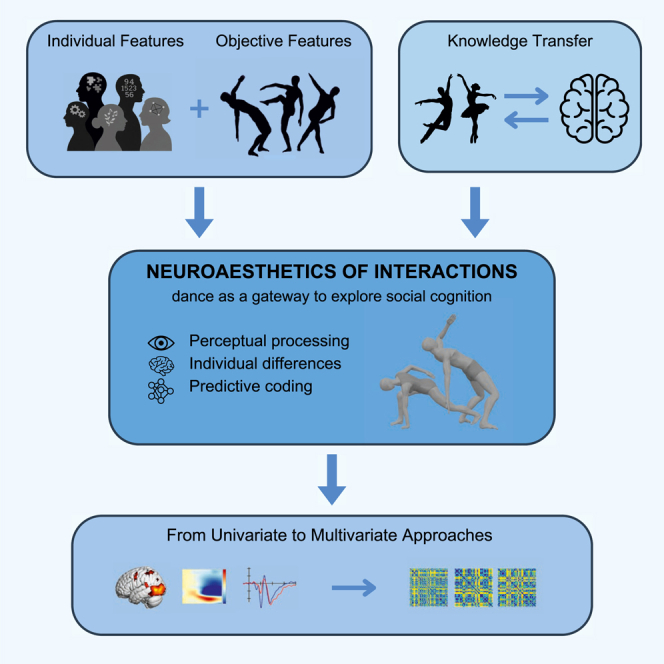

## Introduction

This review provides an overview of key contributions to the field of visual aesthetic evaluation of the human body and its movements, focusing on studies measuring behavioral and neural responses related to body aesthetics.^1^ Specifically, we aim to integrate insights from research on the neural systems involved in the aesthetic evaluation of single-body forms and movements with findings from studies on the neural mechanisms underlying the perception of groups of individuals moving together, as initiated by Vicary et al.^2^ This shift introduces a novel perspective we call the “*neuroaesthetics of interactions*”, proposing that the aesthetic evaluation of interpersonal interactions may involve an interplay of neural systems supporting visual, sensorimotor, and reward processing, as well as neural systems that support higher-order social functions (e.g., empathy and Theory of Mind). This area of research is closely related to developments in social neuroscience and offers valuable insights for studies of social cognition in both healthy and clinical settings. It is also relevant for fields such as empirical aesthetics, human-robot interaction, and the performing arts.

To this end, the review integrates correlational evidence (e.g., electroencephalography – EEG, functional magnetic resonance imaging – fMRI, functional near-infrared spectroscopy – fNIRS) and causal evidence (e.g., transcranial magnetic stimulation – TMS, transcranial direct-current stimulation – tDCS, and studies involving patients with cerebral and spinal lesions) on the neural underpinnings of single-body form and movement processing. It connects these with new findings on behavioral and neural responses associated with interpersonal interaction perception and highlights the roles of both objective^3^ features of stimuli and subjective^4^ characteristics of the observers in aesthetic evaluation. We then discuss recent findings related to the aesthetic evaluation of multiple individuals moving together, particularly in relation to movement timing and synchrony, and suggest that dance may offer a fruitful context to study the aesthetics of interactions. Finally, we critically assess additional factors that may influence the aesthetic appraisal of interacting bodies beyond movement timing, emphasizing the potential of dance as a promising framework for investigating interpersonal interaction aesthetics.^5^^,^^6^

In recent years, the application of neuroscientific methods to understanding the emotional, cognitive, and neural responses to dance movement repertoire has paved the way for more refined investigations into movement aesthetics.^7^^,^^8^^,^^9^^,^^10^^,^^11^^,^^12^^,^^13^ Dance enables comparisons between observers with varying levels of motor and observational expertise, as well as between kinematically similar gestures from different technical repertoires. The opportunity to focus on specific aspects of physical movement—such as timing, emotional expression, and communication—has encouraged scientists to engage more directly with the performing arts field.

We begin by reviewing the neural systems involved in body form and movement visual perception (section visual perception of the human body form and movement), given that many studies have explored the extent to which the aesthetic evaluation of the human body and its movements relies on systems that support the visual perception and representation of body-related stimuli in somatic, motor, and visceral networks (section from perception to aesthetic evaluation). In section the dance framework for movement aesthetics, we discuss how this literature connects to action understanding, describing the links between movements, actions, and dance, and review the rich body of work on the aesthetic evaluation of dance. Section from single-body to two-body and multiple-body interactions focuses on the neurocognitive mechanisms underlying the visual perception and aesthetic evaluation of two-body and multiple-body interactions and introduces a shift toward a neuroaesthetics of interactions. Finally, in section discussion, we provide a critical discussion of emerging topics around this novel research perspective, as well as open challenges, methodological issues, and potential applications.

## Visual perception of the human body form and movement

### Perceiving the form of the body

It is known that the occipitotemporal cortex (OTC) contains brain regions specialized for processing human body form,^14^^,^^15^^,^^16^ namely, the extrastriate body area (EBA, overlapping with the human middle temporal complex, or hMT+) and fusiform body area (FBA), identified in the early 2000s and confirmed ever since.^17^^,^^18^^,^^19^^,^^20^ Enhanced hemodynamic responses in EBA and FBA have been found in response to images depicting real bodies or body parts, as well as silhouettes, line drawings, and stick figures compared with faces, animals, and objects. Over the last two decades, the central role of these regions in static and dynamic body recognition has been confirmed by converging evidence from studies using different methodological approaches. TMS investigations have shown impaired performance in a matching-to-sample task in response to bodily stimuli (but not faces or objects) after repetitive stimulation of EBA (but not V1), suggesting a causal relationship between EBA activity and the visual processing of the form of the human body or its parts.^21^^,^^22^^,^^23^^,^^24^^,^^25^ Recently, TMS applied over EBA during the presentation of a pre-target verbal cue was found to reduce the validity effect in response to bodies but not scenes, indicating the role of pre-stimulus activity in EBA for expressing perceptual expectations about the human body.^26^ Lesion studies have corroborated the notion that the activity of not only the EBA^27^ but also the FBA is necessary for body form discrimination.^28^ Moreover, the activity of the EBA has been studied not only in relation to perception but also to the aesthetic evaluation of body images. Evidence suggests that the activity of this region is sensitive to the aesthetic evaluation of bodily stimuli and may shape aesthetic sensitivity.^7^^,^^8^^,^^29^ This will be further reviewed in sections neuroaesthetics of the body form and dance aesthetics: the role of visuomotor processing and expertise.

Body image processing is typically associated with modulations of electrophysiological markers, including the occipitotemporal N190 component elicited by stimulus observation (ERP, event-related potential^30^) and increased power (ERS, event-related synchronization) in the theta frequency band during body part categorization tasks (4–7 Hz^31^^,^^32^). Evidence from MEG studies^33^ and intracranial recordings^34^^,^^35^ has localized the neural generators of such markers within EBA. While there is evidence of N190 modulation as a function of emotional body language^36^ and body orientation,^37^^,^^38^ the impact of pleasantness on early visual processing stages remains poorly explored.^39^^,^^40^ Additionally, since neural oscillations are believed to support specific mechanisms for inter-area communication^41^^,^^42^ and information transfer, it is noteworthy that no study has yet evaluated whether specific frequencies might support inter-area communication mechanisms that mediate the aesthetic evaluation of body images.

At a behavioral level, body perception also appears to benefit from configurational processing, similar to face perception.^43^ At a neural level, however, while FBA shows a preferential response to whole-body presentation,^44^ evidence for both configurational^45^ and local (body parts^18^^,^^46^) body processing in EBA has been reported. This debate is associated with understanding the neural bases of the body inversion effect (BIE)—a phenomenon in which recognition performance decreases when the human body is presented upside-down compared to its natural, upright orientation.^47^ The BIE typically manifests as reduced accuracy and increased reaction times, alongside an increased N190 response to the inverted (as opposed to upright) body^37^ or slower access to conscious vision in binocular rivalry setups.^48^ This evidence has been interpreted as an index of impaired configurational processing due to body rotation, an effect previously reported for face but not object.^49^ Supporting this view, a recent EEG study indicates that increased attentive selection processes (e.g., selection negativity/posterior N2 component) are required to categorize a body when it is not in its canonical orientation.^38^

As discussed in the following sections, fundamental questions in the field of neuroaesthetics—and even more so in the neuroaesthetics of body form—are: To what extent are sensory-specific visual systems involved in modulating individuals’ aesthetic evaluations of body images? How does acquired experience plastically modulate the morphological organization and functional response of these systems to body images? Finally, do top-down influences modulate the engagement of these visual systems during the perception and evaluation of body images—and if so, through what mechanisms?

### Perceiving the dynamic body: Actions and complex movements

The observation of body movements and actions, even without clear form information,^50^^,^^51^ engages multiple OTC areas. Different neuron populations within the OTC may be variably attuned to body form and motion information.^16^ Regions such as the EBA and the FBA respond to both the static body shape, body postures implying movements, and dynamic body and body parts,^15^^,^^36^^,^^52^ while areas like the occipital face area (OFA) and fusiform face area (FFA) are engaged during the processing of both static faces and facial movements.^53^^,^^54^^,^^55^ The posterior superior temporal sulcus (pSTS) shows sensitivity to biological (compared to non-biological) motion, as revealed by the observation of point-light animations (i.e., movements represented by a set of point-light dots displaying human figures versus scrambled animations).^50^^,^^56^ Notably, the STS, which has dense connections with the amygdala,^57^^,^^58^^,^^59^^,^^60^ plays a critical role in encoding social and emotional cues from dynamic and implied, socially relevant, body movement.^51^^,^^61^^,^^62^^,^^63^^,^^64^^,^^65^ Supporting this view, the STS also underlies the conscious perception of emotional body postures^66^ and motion.^67^ A recent fMRI study^68^ involving multivoxel pattern analysis (MVPA) showed movement-specific representations (e.g., walk, run, jump, skip) in the EBA, hMT+, and pSTS, and identified functional connections between the EBA and several regions in the broader fronto-parietal Action Observation Network (AON; see below; e.g., IFG, IPL, precentral gyrus, middle and superior temporal gyri, lateral OTC, precuneus, and cerebellum). As a whole, such pieces of evidence seem consistent with the view that the OTC supports lower-level feature processing,^69^ as part of the visual ventral stream, with subsequent integration of information into a broader cortical network (i.e., AON), which underpins action simulation for anticipation and understanding.^70^

During the observation of body movements and actions, the AON, comprising temporal, frontal (premotor), parietal, and cerebellar regions,^71^^,^^72^^,^^73^^,^^74^^,^^75^^,^^76^^,^^77^ is activated alongside the OTC (e.g., EBA and STS). The functional role of different AON nodes in movement perception and action understanding remains debated, with competing models proposing vision-based, motor-based, and hybrid approaches.^71^^,^^78^^,^^79^^,^^80^^,^^81^^,^^82^^,^^83^^,^^84^^,^^85^^,^^86^^,^^87^^,^^88^^,^^89^ While motor- or simulation-based theories suggest that transforming visual body movement information into a motor code (e.g., through the activity of the human mirror neuron system) is essential for understanding their meaning, other authors have proposed that the OTC may play a central role in action categorization^90^^,^^91^^,^^92^^,^^93^^,^^94^ and even in action preparation.^95^ The involvement of the OTC in action categorization is supported by evidence of somatotopic-like functional connectivity with regions that underlie cross-modal representations of both observed and performed actions (e.g., in the left postcentral gyrus/anterior parietal cortex^18^^,^^96^). Furthermore, OTC activity has been linked to early access to abstract action representations,^97^ and a recent MVPA study on hand perception suggests a topographic organization in the lateral OTC with selective territories responding selectively to distinct actions.^98^

Relevant to this review is the distinction in OTC regions for social versus transitive actions, represented in the dorsal and ventral portions of the lateral OTC, respectively, and the posterior-to-anterior organization of concrete (object-related) and abstract (transitive and social) action features. A recent EEG study also indicated that hand gestures with social content modulate early visual processing stages.^99^ Specifically, hand images in a shaking posture elicited an increased early posterior negativity (EPN) compared to grasping or still hand postures, suggesting enhanced visual attention toward socially salient gestures. MVPA findings further indicated an above-chance classification of shaking versus grasping hand postures around 150 ms over occipito-parietal sites. Additionally, a recent brain stimulation study provided causal evidence of the left EBA involvement in processing two-body social interactions,^100^ suggesting a confluence of visual, motor, and semantic action information within the OTC^91^ and further fueling the debate on the specific role of AON areas in action encoding. Further research may help clarify whether seemingly opposing views on the role of sensorimotor simulations in the frontoparietal network for movement prediction and action understanding could, in fact, offer complementary insights.^89^

In the last decades, a predictive coding framework for action understanding grounded in empirical Bayesian inference has been proposed.^101^^,^^102^ According to this account, minimizing prediction errors through reciprocal interactions across hierarchical cortical levels (representing intention, goal, motor program, and kinematics) enables action understanding. In this framework, comparing predicted and perceived movement kinematics results in prediction errors, which serve to update either action prediction or the motor command. This model aligns with evidence showing that AON activity varies with the familiarity and novelty of observed movements^103^ and with increased somatotopic excitability in the motor system in response to unexpected or erroneous movements.^104^^,^^105^ Such a framework is also relevant for aesthetic evaluation of movements, which often hinges on surprise and expectation violations.^106^^,^^107^^,^^108^^,^^109^

## From perception to aesthetic evaluation

Over the past few decades, an increasing number of neuroscientific studies have explored the neurobiological mechanisms underlying our aesthetic evaluations of various artistic or natural stimuli. Neuroaesthetics seeks to provide empirical aesthetics with methods, tools, and a conceptual framework based in cognitive neuroscience.^110^^,^^111^^,^^112^^,^^113^ This approach allows for the investigation of how sensory experiences are triggered by a stimulus, linking its properties to the neural activity involved in its perception and aesthetic evaluation^111^^,^^114^^,^^115^—what Fechner, more than a century ago, referred to as Outer and Inner Psychophysics.^116^

As will become apparent throughout this review, the majority of neuroscientific studies on body and movement aesthetics involve aesthetic evaluation tasks. Participants are typically asked to evaluate images or videos using various rating scales (e.g., likability, enjoyment, interest, beauty, and emotional valence), while their physiological (e.g., heart rate and skin conductance) or neural responses (e.g., through EEG and fMRI) are recorded or manipulated (e.g., via non-invasive brain stimulation). By systematically changing specific features of the stimuli and observing parametric changes in neurophysiological activity and subjective ratings,^9^^,^^117^ it becomes possible to deconstruct a complex phenomenon (e.g., dance) into simpler, quantifiable elements and assess their relative contributions to aesthetic evaluation (e.g., temporal features^118^).

Although aesthetic evaluation is not limited to artistic stimuli, the idea that visual art appraisal is based on the organization of the visual brain was first proposed by Semir Zeki.^119^^,^^120^ Extensive evidence has since then confirmed that activity in different visual areas is modulated during the aesthetic evaluation of paintings, depending on both low-level (e.g., color, shape, and motion) and high-level (e.g., semantic content: presence of a face, a body, or a landscape) features.^115^^,^^121^^,^^122^ Of particular relevance to this review are studies on the neurocognitive substrates underlying the evaluation of bodies, body parts, and movement^7^^,^^123^^,^^124^ in terms of their beauty and likability. In the sections that follow, we argue for a framework in which aesthetic evaluation depends on the interplay between objective stimulus features, individual traits (e.g., experience and motivation), and sociocultural background.^125^

### Neuroaesthetics of the body form

The involvement of occipitotemporal stimulus-specific visual areas in the aesthetic evaluation of body stimuli has been supported by several neuroimaging and brain stimulation studies.^8^^,^^23^^,^^29^^,^^126^^,^^127^^,^^128^^,^^129^ This aligns with previous findings on the role of visual areas in the aesthetic evaluation of faces^130^ and non-body artworks.^29^^,^^121^^,^^122^^,^^131^^,^^132^^,^^133^^,^^134^^,^^135^^,^^136^ For example, in an fMRI study by Di Dio et al.^129^ participants evaluated images of sculptures that complied (vs. did not comply) with canonical proportions between body parts (based on the golden ratio 1:1.618) for their beauty and symmetry. Higher activations for canonical (vs. manipulated) sculptures were found in lateral occipital areas (EBA), as well as in the right insula, prefrontal areas, and precuneus. In a subsequent study, the same authors compared artistic (i.e., sculptures) and natural (i.e., photographs of real bodies) body images, showing similar activity in occipitotemporal visual areas (EBA), frontoparietal (e.g., IPL, vPM, and IFG), and subcortical regions (e.g., amygdala and hippocampus).^127^ Statues, as compared to real bodies, also elicited stronger activity in visual areas (e.g., fusiform gyrus) and the right anterior dorsal insula, while real bodies more strongly activated the STS. These results were interpreted as evidence that the aesthetic evaluation of body images involves the visual processing of specific features (e.g., proportions) and may also recruit sensorimotor and affective systems.^128^

Cazzato et al.^126^^,^^137^ provided causal evidence for the contribution of EBA to body aesthetics, modulated by gender congruence between the observer and the observed body. rTMS (repetitive TMS) applied over the right EBA (compared to the vertex) increased liking for different-gender bodies in female participants.^137^ However, rTMS over both right and left EBA decreased liking for different-gender bodies in male participants, suggesting gender-related differences in hemispheric asymmetry of EBA in body aesthetics. In a subsequent study,^126^ the same research group replicated the increased liking for different-gender bodies after rTMS over EBA, and additionally reported reduced liking for same-gender bodies following rTMS over the dorsal premotor cortex (dPM). The authors suggested two potentially complementary explanations for their results. The contributions of motor and visual areas in body aesthetics could reflect the processing of different aesthetic properties of the stimulus (e.g., implied motion vs. body form), but could also reflect differences in sensorimotor embodiment of same-gender vs. different-gender bodies.

These results are consistent with a meta-analysis of 56 neuroimaging studies, which reported consistent engagement of occipitotemporal, frontoparietal (e.g., IPL and IFG), insular areas, and subcortical structures (e.g., amygdala), along with the orbitofrontal cortex, during aesthetic evaluation of visual stimuli.^138^ Altogether, this body of work support the notion that, when perceiving body-related stimuli, aesthetic evaluation involves not only visual areas processing form (e.g., EBA) and implied motion (e.g., EBA, hMT+, and STS), but also motor regions (e.g., IPL and IFG) that contribute to sensorimotor transformations and embodiment processes.

### Neuroaesthetics of the dynamic body: The role of motor simulation

Inspired by the wave of theories emphasizing a central role of sensorimotor mechanisms in higher-order cognitive functions,^139^^,^^140^ one of the milestones in the field of neuroaesthetics is the so-called *embodied simulation account of aesthetics* proposed by Freedberg & Gallese.^141^ The authors suggest that two main components are involved in (visual) art evaluation: the observer’s embodied empathetic responses to an artwork’s representational content and the simulation of the visible traces of the artist’s actions. According to this theory, visuomotor simulations enable the observer to feel bodily engaged with observed actions allowing them to identify with emotions and empathize with bodily sensations. Similar mechanisms also allow for the simulation of the artists’ gestures required to create the artwork, as seen in the abstract works by Jackson Pollock and the cut canvases by Lucio Fontana.^142^ These automatic empathic responses are proposed as a fundamental level of engagement with artworks, facilitating a direct, experiential understanding of the depicted intentions and emotions through embodied simulation processes.^141^

Evidence linking the observer’s motor system activity and their aesthetic evaluation has been supported by studies using paintings. For instance, when participants perform hand movement tasks before evaluating abstract paintings, they show a preference for paintings created with congruent (vs. incongruent) hand movements, such as pointillist-style versus stroke-style^143^^,^^144^). A recent TMS study assessed corticospinal excitability during the observation of visual stimuli and found stronger excitability in the muscle needed to reproduce the painting style (i.e., wrist extensor)—while participants viewed paintings. This muscle-selective facilitation mediated observers’ lower liking ratings and correlated with individuals’ empathy dispositions.^145^ One interpretation is that covert simulation of the artist’s movements may influence the aesthetic evaluation.^141^ However, this view is not without criticisms. Scholars argue that high-level cognitive processes do not influence perception (see, for example, Firestone & Scholl, 2015^146^) and should not be considered as contributing to aesthetic evaluation. These accounts propose that any apparent top-down influence on perception can be attributed to more general “pitfalls”, such as task demands, low-level differences, and peripheral attention. The extent to which top-down cognitive factors influence perception and aesthetic evaluation remains an important question for further research.

The possible role of motor system activity in movement evaluation has also been studied in the context of “non-artistic” movements, such as typing actions,^147^ eye movements,^148^ and interactions with everyday objects.^149^ Converging evidence indicates that movements that are practiced, and thus more familiar and feasible for the observer, are generally preferred over unfamiliar ones—likely due to increased processing fluency.^11^^,^^150^ Interestingly, the effect appears to reverse for more complex and artistic movements, such as dance (see section dance aesthetics: the role of visuomotor processing and expertise on Dance aesthetics). For example, Cross et al.^9^ observed increased activity in the bilateral OTC and right inferior parietal lobule (IPL) while non-expert participants watched dance movements that they rated as more likable but found difficult to reproduce. This negative relationship between movement reproducibility and evaluation was confirmed in a subsequent study^118^ and aligns with the effort heuristic account,^151^ which suggests a preference for artworks perceived as effortful and difficult to produce. Differences in aesthetic evaluation between artistic and non-artistic movements may depend on multiple factors, including the aesthetic intent of the movements, their kinematic complexity, the body parts involved, and the observer’s experience with similar movements. Future research is needed to systematically investigate these variables, given the speculative nature of these observations.

As discussed throughout this review, researchers use different approaches to operationalize movement (e.g., dance) aesthetics for neuroscientific study. These include varying task instructions (e.g., how much did you enjoy/like watching this video?), stimulus type (e.g., images, videos, and point-light animations), and measurement techniques. Subjective evaluations (e.g., on Likert scales^118^) are typically associated with brain activity measures such as EEG^152^ and fMRI,^9^ or modulated via brain stimulation protocols like TMS.^8^^,^^153^ Subjective ratings have also been linked to physiological changes, including skin conductance, heart rate, and pupil dilation.^154^^,^^155^^,^^156^ More recently, experimental approaches have begun investigating neural and physiological responses during live dance performances, often with larger audiences in real theater settings.^157^ This shift represents a critical step toward understanding the neuroaesthetics of interactions and performative art making.

### Reward system and emotional responses to movements

In addition to the involvement of sensory-specific systems and the fronto-parietal system in the sensorimotor transformation of observed body movements, the activity of cortical and subcortical reward systems is frequently described. Kirsch et al.^1^ proposed that during movement observation, the interplay between the visual, sensorimotor, and reward systems contributes to the aesthetic experience of the observer. This view aligns with the aesthetics perception model proposed by Nadal et al.,^158^ which outlines three major cortical networks involved in positive aesthetic experiences: (1) circuits supporting top-down processing and evaluative judgment (e.g., attentional modulation^159^), (2) low- and high-level sensory circuits for processing perceived stimuli, and (3) circuits within the reward system, including cortical (e.g., orbitofrontal cortex^117^^,^^136^) and subcortical (e.g., ventral striatum, caudate nucleus, substantia nigra, and amygdala^138^) areas.

Most studies on movement aesthetics have focused on the first two networks in stimuli evaluation—particularly regions associated with sensorimotor processing,^7^^,^^160^ attention,^161^ and error anticipation^162^^,^^163^—as modulated by the observer’s expertise. However, the conditions under which the third network—the reward system—is recruited during the aesthetic evaluation of bodily stimuli remain less well understood. While previous studies have linked the activity of the dopaminergic reward system^164^ to the pleasure associated with listening to music^165^^,^^166^ and viewing visual arts,^117^^,^^136^ only a few studies have explored this link in the context of body and movement aesthetics.

For instance, Jang & Pollick^167^ found enhanced early processing of familiar movements in the right temporoparietal junction (TPJ) and left retrosplenial cortex (RSC) when experienced viewers and ballet dancers observed dance. At the same time, dance observation seems to engage somatosensory processing in dancers’ right somatosensory and bilateral motor areas. Additionally, regions typically associated with Theory of Mind tasks (e.g., right orbitofrontal cortex and TPJ) were modulated in expert observers. These findings are noteworthy because brain networks underlying higher social functions—such as the ability to attribute mental states to oneself and others^168^^,^^169^—may also play an important, complementary role in the aesthetic evaluation of social interaction.

A meta-analysis by Brown et al.^138^ compared brain activity from 93 neuroimaging studies examining positive-valence aesthetic evaluation across four sensory modalities: vision, audition, gustation, and olfaction. Common areas of activation included the orbitofrontal cortex, anterior cingulate cortex, anterior insula, and ventral regions of the basal ganglia. A conjunction analysis across all four modalities identified a cluster in the right anterior insula as a potential supra-modal area for processing positive-valence aesthetics. This region is typically involved in emotion, empathy, sensory, and visceral processing. The authors proposed a model in which valence appraisal depends on recurrent connectivity between the anterior insula (involved in awareness of the observer’s homeostatic state) and the orbitofrontal cortex (involved in exteroceptive perception of the object).

More recent studies have reported engagement of reward-related regions in response to both low-level features and higher-level semantic meanings of movements. For example, increased activity in the nucleus accumbens and subthalamus has been observed for dance sequences rated as more likable.^170^ The nucleus accumbens was also activated during the anticipation of social reward conveyed through body movements as compared to written texts.^171^ Additional findings have demonstrated the reward value associated with biological motion perception,^172^ revealing enhanced activity in the anterior cingulate cortex, caudate, and nucleus accumbens, along with superior temporal, parietal, and postcentral areas, in response to positive (vs. control) body movements used as incentive feedback.^171^

Further studies are needed to clarify how reward value and emotional response interact in the aesthetic evaluation of movement. This includes accounting for both the objective stimulus features (e.g., postures and movements) and the observer’s subjective reactions (e.g., liking and emotional evaluation), some of which are described throughout this manuscript. Understanding how the reward system contributes to aesthetic evaluation also requires addressing open questions—such as the role of positive and negative emotions,^173^^,^^174^^,^^175^ and the influence of expectations and surprise^176^^,^^177^^,^^178^^,^^179^—particularly in the context of interactions.

In the following sections, we describe why dance provides an ideal context for studying the perceptual, emotional, cognitive, and contextual cues that shape the aesthetic evaluation of interactions.

## The dance framework for movement aesthetics

Dance, as a distinctive category of human movement repertoire, can be defined as a universal form of human expression^180^ that emerges from creative processes involving the movement of human bodies through time and spatial layouts.^181^ One aspect that makes dance unique compared to other art forms (such as music-making) or linguistic exchanges is that the dancer’s body serves as both the subject (e.g., the tool creating the artwork) and the object (e.g., the perceived artwork). In the case of music, the musician’s body is crucial when interacting with an instrument to create a piece, but it is not central to the listener’s experience of the musical work. In contrast to acting, the semantic meaning in dance does not rely on verbal language, meaningful actions, or mimicry. Instead, it depends on both “what” is communicated and “how” it is conveyed through the moving body.^182^

Dance is inherently linked to effective non-verbal communication via observed movement.^183^^,^^184^ Ideas, emotions, and stories in dance are conveyed by modulating various movement elements, including their organization in space (e.g., directions, levels), rhythm (e.g., tempo, duration, and accent), dynamics (e.g., force and relaxation), shapes, gestures, and motifs.^185^ In different terms, dance is a discipline that combines athletic skills and refined motor control with artistic expressivity. As such, it is a valuable activity for neuroscientific research on expressive and perceptual functions, spanning topics from movement kinematics and skill acquisition to choreographic structures and the transmission of meaning and emotional content (for further insights into dance in empirical aesthetics and neuroaesthetics, see Christensen et al., 2017^186^; Christensen & Calvo-Merino, 2013^187^; Cross & Ticini, 2012^188^; Cross & Orlandi, 2020^189^).

**Figure 1:**
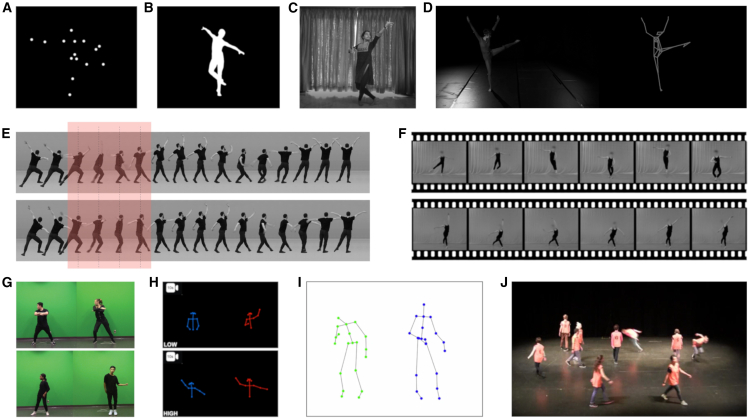
Example of stimuli used in previous investigations on movement aesthetics (A) Point-light animations depicting a dancer performing movement sequences portraying different emotional states, a still image taken from one of the videos (© 2022 Smith, Cross^194^; published by Springer Nature; licensed under CC-BY 4.0). (B) The silhouette of a dancer performing movement sequences portraying different emotional states, a still image taken from one of the videos (© 2023 Christensen, Bruhn, Schmidt, Bahmanian, Yazdi, Farahi, Sancho-Escanero, Menninghaus^195^; published by Springer Nature; licensed under CC-BY 4.0). (C) A dancer performing movement sequences from Bharatanatyam Classical Indian dance, a still image taken from one of the videos by Darda & Cross^196^ (© 2023 Darda, Cross; published by Elsevier Ltd.; licensed under CC-BY 4.0). (D) A still image from the choreography presented by a real dancer and a stick figure by Poikonen et al.^161^ (© 2018 Federation of European Neuroscience Societies and John Wiley & Sons Ltd). (E) Static frames from a dance sequence to highlight the difference in kinematic complexity (varied on top, uniform on bottom) between two versions of the same sequence (adapted from the original, © 2020 Orlandi, Cross, Org^118^; published by Elsevier B.V.; licensed under CC-BY 4.0). (F) Static frames from two dance sequences (adapted from the original, © 2021 Christensen, Azevedo, Tsakiris^197^; published by Elsevier B.V.; licensed under CC-BY-NC-ND 4.0). (G) Pairs of dancers performing hip hop sequences in a synchronously (top) or asynchronously (bottom), a still image from two of the videos (adapted from the original, © 2020 Tang Poy and Woolhouse^154^; published by Frontiers; licensed under CC-BY 4.0). (H) Stick figures depicting dyads moving in a high vs. low synchrony, a still image from two of the videos (adapted from the original, © 2024 Moffat, Cross^198^; published by Springer Nature; licensed under CC-BY 4.0). (I) Point-light animations depicting dyadic configurations created by instructing the participants to freely move (dancing) on musical stimuli (adapted from the original, © 2019 Hartmann, Mavrolampados, Allingham, Carlson, Burger, Toiviainen^199^; published by Springer Nature; licensed under CC-BY 4.0). (J) Group Study (2015) by Matthias Sperling, video still, from Vicary et al., 2017^2^ (© 2017 Vicary, Sperling, von Zimmermann, Richardson, Orgs; published by PLOS; licensed under CC-BY 4.0). A copy of the CC-BY 4.0 License can be found at http://creativecommons.org/licenses/by/4.0/. A copy of the CC-BY-NC-ND 4.0 License can be found at https://creativecommons.org/licenses/by-nc-nd/4.0/.

Dance provides valuable insights into multisensory neural representations of movements^6^ and the mechanisms associated with its evaluation in terms of emotions, semantics, and aesthetics.^188^ Dance stimuli can be used to investigate expertise-related plastic changes in the brain,^190^ motor control mechanisms,^191^ non-verbal communication,^183^ motor learning,^192^ and memory.^193^ They also are instrumental in exploring how objective features (e.g., timing, fluency, and symmetry) of movement engage specific brain networks and shape our aesthetic perception of movement and dance. Figure 1 provides examples of dance stimuli used in movement aesthetic research, illustrating the diversity of images and videos used by different authors. These include images of complex body postures, short video clips of dance steps, point-light animations, stick figures, and extended video recordings of full dance sequences.

Building on our understanding of the neurocognitive mechanisms involved in body and movement perception, as well as the evidence reviewed earlier, we summarize in Box 1 existing studies that illustrate the advantages of using dance as a framework for cognitive neuroscientific research beyond neuroaesthetics. Here, we outline the main features that make dance an effective and fertile field for investigating interaction aesthetics at various levels.

(1) Dancers are trained in different techniques and styles, each characterized by specific rules and principles. This shared theoretical and practical knowledge allows for comparisons between expert and non-expert participants,^162^ as well as between kinematically comparable yet distinct dance repertoires (e.g., ballet vs. capoeira^4^).
(2) The repertoire of dance gestures is broader than in sports (e.g., shoots in basketball or football), and especially in modern and contemporary dance, it is potentially limitless (e.g., the creation of novel movement, choreographic languages, or “quality of movement”^200^^,^^201^).
(3) Dance movements are non-object-directed and non-tool-mediated (i.e., intransitive) and lack universally recognized symbolic meanings. They may be performed in isolation or in conjunction with context (e.g., music^161^).
(4) Dance features grammatically structured movements^11^ that can be analyzed as single units^202^ or as components of more complex sequences.^118^
(5) Dance can be performed and observed at both the individual (e.g., solo) and interactive (e.g., pas de deux and ensemble)^2^^,^^203^ levels, making it relevant for studies of joint actions—social interaction in which two or more individuals coordinate their actions in space and time to achieve a common goal.^204^
(6) The choreographic process can be conceptualized as an information transfer encoded in movement kinematics^183^ from a sender (e.g., dancer/choreographer) to a receiver (e.g., audience), allowing for detailed investigation into the link between non-verbal communication, emotional body language, and kinematics.^205^

Box 1 **Examples of the advantages of using dance as a framework for neuroscientific studies**

**Table:** 

Advantages of dance	Examples and corollaries
Different techniques and styles	• Each technique and style have rules and principles (e.g., ballet, contemporary, Bharatanatyam)^202^^,^^206^ • Differences and similarities between physical and cognitive processes underlying expertise acquisition^207^ • Shared physical and theoretical knowledge between experts^208^ • Comparability between kinematic similar movements from different repertoire^4^ • Between-group investigations (e.g., experts vs. non-experts)^202^
Repertoire of movement	• Creation of novel movements (e.g., in contemporary dance)^194^ • Creations of movement variations^209^
Non-symbolic movements	• Dance movements as intransitive actions (e.g., non-object directed, non-tool mediated) and lacking any universally recognised symbolic meanings • Movements considered per se or integrated into a broader context of music/sound, costumes, and space^161^
Movement grammar	• Dance movements are units that can be considered in isolation^7^ • Dance movement can be merged to create complex sequences following rules^118^^,^^210^ • Possibility to introduce errors or variations based on the technique considered^162^
Number of dancers	• Movements performed by a single dancer (e.g., solo)^9^ • Movements performed by two dancers (e.g., duo)^154^ • Movements performed by a group of dancers (e.g., ensemble)^211^
Communication	• Choreographic process as an information transfer process^183^ • Relationship between the choreographer (e.g., creator), dancer (e.g., executor), and audience (e.g., receiver)^183^ • Possibility to focus on physical features of movements^212^ • Possibility to focus on emotional and semantic features of movements^156^ • Relationship between movement kinematics, emotional body language, and non-verbal communication^205^ • Dance can be perceived live or via video recording (e.g., liveness)^157^ • Dance can be perceived as a social phenomenon (e.g., single observes vs. group of observers)^213^

Given these strengths, it is not surprising that a growing number of studies are focusing on the aesthetic evaluation of the body and movement using dance as a central tool. While the discussion section of this review will address how these advantages apply to the study of two-body interaction aesthetics, we will also consider key limitations that must be addressed to optimize the use of dance as a research framework.

### Definitions and consistency of the terminology in movement neuroaesthetics

Before delving into the specific factors involved in the aesthetic appraisal of body and movements’ visual appearance in dance, it is important to highlight that the current literature is characteriszed by a wide variety of terminology, definitions, and methodological approaches. For instance, there is a marked heterogeneity in how scholars conduct their studies—evident, for example, when comparing inclusion criteria for expert (vs. non-expert) participants (as noted by Swann et al. in the field of sport expertise^214^), the tasks used to measure aesthetic appraisal (e.g., likability, enjoyability, beauty, or interest), and the rationale for selecting particular movement repertoires as stimulus material.^215^

A more subtle but crucial issue concerns the (dis)agreement on the relationship and boundaries between movements, actions, joint actions, and dance. Different authors using dance stimuli for aesthetic research refer to these stimuli in various ways, reflecting the diversity of movement types and complex body postures employed. Terms such as biological motion,^209^ movements,^152^ dance actions,^216^ steps,^202^ ballet moves,^4^ gestures,^212^ sequences,^170^ joint actions,^2^ and dance-like actions^217^ have all been used. The presence or absence of shared goals, meanings, and transitivity helps to distinguish different movement and action categories (e.g., imitation of meaningful vs. meaningless movements),^218^ distinctions that are also reflected at the neural level. The question of when and how a movement becomes an action—or when a sequence of movements becomes dance—depends both on the researcher’s theoretical framework and on the perceiver’s familiarity with the movements. Progress toward a neuroaesthetics of interactions will require a coordinated effort among researchers from multiple disciplines (e.g., neuroaesthetics, social cognition, social and affective neuroscience) to establish shared terminology and methodological standards.

In this review, we use the term “aesthetics of movement” broadly to emphasize the intransitive nature of dance (i.e., not object-directed or tool-mediated, though it may involve a partner’s body) and its lack of universally recognized symbolic meanings, especially for non-expert observers. The more visual and/or motor expertise one gains with a movement repertoire (e.g., ballet), the more motor programs they acquire to reproduce those movements, along with the theoretical knowledge of how they are executed and named (e.g., “pas de bourrée” and “grand jeté”).^206^ When combined according to defined rules, these movements act as grammatical elements, forming more complex sequences,^208^^,^^219^ that ultimately convey messages to the audience through non-verbal communication.^183^

Non-experts lack theoretical, semantic, and motor knowledge underlying such repertoires; instead, they are more likely to focus on movement’s kinematic features, which are believed to mediate emotional and aesthetic responses.^205^^,^^220^ These features include low-level elements such as speed, amplitude, balance,^5^ as well as perceived effort^221^^,^^222^ and reproducibility.^9^

As Hagendoorn has pointed out, there are significant similarities between dance and verbal language at the syntactic level, though not at the semantic level (e.g., no true/false prepositions or first-order logic).^223^ In terms of syntax, parallels between dance and language are supported by studies investigating the segmentation of dance sequences^210^^,^^224^^,^^225^ and the perception of error or variations.^162^^,^^209^ When observers are asked to divide a movement sequence into individual units, experts tend to segment them into larger units than non-experts—suggesting that motor experience influences how movements are processed and grouped.^210^

Several neuroimaging studies have also revealed shared structural mechanisms in the processing of dance (and music) and that of language.^224^^,^^226^ However, whether dance sequences and language share common neurocognitive processing remains an open question, one that calls for further investigation from a multidisciplinary perspective.

In contrast, strong evidence supports a robust relationship between acquired visuomotor expertise, motor simulation, and aesthetic evaluation of movement, as revealed by numerous studies. This relationship will be explored in detail in the following paragraphs.

### Dance observation: Motor simulation and visuomotor expertise

As with musicians^227^^,^^228^ and athletes,^229^^,^^230^ dancers show structural, functional, and connectivity-related neural modifications (for a review on dance, see Karpati et al., 2017^190^). Motor skill acquisition is associated with plastic changes across several brain networks, likely underlying specific enhancements in various neurocognitive domains,^231^ such as perception, motor control, memory, and emotion recognition. Experts also demonstrate an enhanced ability to perceive macroscopic errors^162^ and subtle movement variations.^209^ These abilities may arise from theoretical knowledge, refined motor imagery^221^^,^^222^^,^^232^ and anticipatory action-outcome simulation.^104^^,^^233^^,^^234^^,^^235^

In a seminal study by Calvo-Merino et al.,^216^ the authors compared brain activity in expert ballet and capoeira dancers and non-experts while they watched videos of ballet and capoeira movements. Greater engagement of the bilateral premotor and intraparietal sulcus (PM and IPS), right superior parietal lobule (SPL), and left posterior superior temporal sulcus (pSTS) was found when experts observed movements from their own dance repertoire, compared to kinematically similar but untrained actions. This study provided the first evidence of frontoparietal activity reflecting action representation via simulative mechanisms in response to complex movements like dance. In a subsequent study,^4^ the authors further dissociated the contribution of visual and motor expertise to the activation of sensory (visual) and motor cortical nodes of the AON during dance observation. By showing male and female ballet dancers a series of actions from both same-gender (entailing motor expertise) and opposite-gender (implying only visual familiarity) techniques, they found enhanced activity within the left PM, bilateral intra-parietal cortex, and cerebellum in response to same-gender (vs. opposite-gender) actions, suggesting that fronto-parieto-cerebellar responses during dance observation reflect a form of motor simulation.

Later studies have provided further evidence that acquired visual experience alone can induce covert simulation of dance movements and that this is linked to the observer’s empathic ability.^10^ Consistent with these early neuroimaging studies, electrophysiological research by Orgs et al.^152^ revealed greater event-related desynchronization in alpha and beta frequency bands in dancers (vs. controls) while observing well-known dance movements—indicating modulation of AON activity due to professional practice. Expertise effects have also been observed during the viewing of extended contemporary dance sequences accompanied by music.^161^ Increased interhemispheric theta (phase) synchrony was found over frontocentral sites in dancers (vs. musicians and laypeople), suggesting more refined processes associated with multimodal processing, spatial attention, and movement time prediction. More recently, a study showed bilateral engagement of the OTC in expert dancers, compared to more asymmetrical, right-lateralized OTC activity (indexed by the N2 component) in controls during observation of movements followed by kinesthetic motor imagery.^202^ Dancers (vs. controls) also showed faster visual processing of movement (indexed by an early temporoparietal P2 component) and greater ease in recognizing technical gestures (indexed by a larger frontocentral P300 component).

Altogether, this body of evidence suggests that dance observation engages different nodes of the AON depending on the observer’s level of expertise and the experimental task.^6^^,^^192^ These findings support the motor simulation account of action perception, in which sensorimotor and kinesthetic, visceral, and somatic simulations contribute not only to movement perception, anticipation, and understanding but also to aesthetic evaluation.

In the next sections, we explore how this differential engagement of visual and motor areas influences emotional and aesthetic responses to dance. We review neuroimaging studies showing the impact of dance expertise on aesthetic evaluation of movement, as well as the involvement of brain regions both within and beyond the AON. We also highlight the complex interplay between stimulus features and individual characteristics that shapes the aesthetic appraisal of dance.

### Dance aesthetics: The role of visuomotor processing and expertise

Causal evidence for the role of occipitotemporal areas in dance aesthetics was provided by a TMS study by Calvo-Merino et al., which showed reduced aesthetic sensitivity to dance postures following selective, transient functional interference with bilateral EBA activity.^8^ The authors suggested that the EBA contributes to the local processing of bodily stimuli during aesthetic evaluations—consistent with earlier neuroimaging findings related to aesthetic evaluation of body form^117^^,^^128^^,^^129^ and dance movements.^7^^,^^9^ In particular, a prior fMRI study by Calvo-Merino et al.^7^ found that non-expert dancers showed greater activity in bilateral visual areas in the medial region and the right premotor cortex when observing capoeira and ballet movements rated as more likable to watch, compared to less likable ones. The authors interpreted this as an indication of a possible contribution of motor resonance processes to the aesthetic evaluation of stimuli involving a motor performance. Similarly, Cross et al.^9^ found increased activity in bilateral OTC and the right IPL while non-expert participants viewed ballet and contemporary dance movements rated as both more likable and more difficult to reproduce. This evidence suggests that movement aesthetics and movement reproducibility are interrelated, and both are supported by the activity of visual and sensorimotor regions.

Studies on short-term acquired visuomotor expertise with dance further support this link between movement aesthetics, reproducibility, and engagement of sensorimotor regions.^160^^,^^170^^,^^236^ In a foundational study by Cross et al.,^160^ expert dancers learned novel short dance sequences over five weeks. At the end of each week, they underwent fMRI scanning while observing both rehearsed and non-rehearsed dance sequences, imagining themselves performing them and evaluating their ability to reproduce them. Physical experience (rehearsed vs. non-rehearsed movement) was associated with increased activity in bilateral STS, left PMv/pars opercularis, supplementary/cingulate motor cortex, and right intraparietal sulcus (IPS). When subjective evaluations of reproducibility were considered alongside physical experience, the left PMv, IPS/IPL, and parahippocampal cortex were selectively engaged for movement that were rehearsed and rated as more reproducible (vs. non-rehearsed and rated less reproducible)—forming a network associated with physical embodiment and action simulation.

The relationship between multimodal experience, reproducibility, and aesthetic evaluation was further investigated in a series of studies by Kirsch et al.^170^^,^^236^^,^^237^ In one study, non-expert participants evaluated dance sequences on dimensions such as likability, interest, complexity, performance enjoyment, and music liking before and after 5 days of training, which varied across three conditions: physical, audiovisual, or auditory training.^236^ A positive correlation between complexity and likability was found before training. After training, only the physical training group showed increased ratings of likability, interest, performance enjoyment, and music liking—supporting the simulation account of aesthetic appraisal by suggesting that aesthetic experience improves with increased visuomotor experience.

In subsequent studies, participants underwent a similar test/retest procedure during fMRI scanning, evaluating dance sequences before and after training. Here, all participants were trained on subsets of dance sequences using three modalities (i.e., physical + visual + audio; visual + audio; audio) to assess how multimodal learning influences perception and affective evaluation through AON modulation.^170^^,^^238^ Results showed a subtle additive effect of training modality on physical performance, associated with increased activations both within (e.g., left PM, left superior temporal gyrus-STG, right intraparietal cortex-IPC) and outside (e.g., anterior cingulate cortex-ACC, posterior cingulate cortex-PCC, anterior fusiform gyrus) the AON. Moreover, while both physical and observational training modulated activity in areas such as the left PM, IPC, superior frontal gyrus (SFG), and PCC, one specific subregion of the left PM was particularly sensitive to the perceived ability to reproduce the observed movements.^238^ When subjective evaluations were included in the analysis (as parametric regressor), increasing likability was linked to a shift of neural activations from subcortical structures (subthalamus and nucleus accumbens) before training to cortical regions (right STG and bilateral STS) after training. The left STG, in particular, was modulated by both sensorimotor experience and increased likability. These results provided evidence that training richness and motor reproducibility influence the aesthetic evaluation of dance via AON modulation.

Another important aspect shaped by long-term training—and likely influencing aesthetic evaluation—is the ability to discriminate emotions expressed through body movement^156^ and to perceive whether two movements are the same or different.^209^ For instance, Christensen et al.^156^ presented expert and non-expert ballet dancers with videos depicting happy and sad ballet gestures, played in their original forward and unusual backward direction, while recording skin conductance. Participants rated how happy or sad the movements made them feel. When gestures were presented in their original forward direction, only experts displayed heightened sensitivity to happy gestures and showed different physiological responses to happy versus sad gestures. Moreover, affective ratings and physiological responses were positively correlated in experts during the forward presentation, suggesting that dance training enhances emotional sensitivity to observed movements.^156^

Similarly, Orlandi et al. showed expert and non-expert contemporary dancers pairs of videos showing novel dance movements while EEG recordings were taken during a secondary task (responding to static body images).^209^ The second video in each pair was either identical to the first (same) one or a slight variation (different). Only the expert dancers automatically detected the variations, as indexed by an enhanced centroparietal N400 component and reduced Late Positivity over centroparietal and frontal regions. Source reconstruction (swLORETA) in the N400 time-window revealed engagement of visual, sensorimotor, and limbic areas—suggesting that dance training modulates visuomotor processing of observed movement.

### Dance aesthetics: The role of individual and objective features

The evidence discussed so far highlights at least two categories of features that contribute to and influence the aesthetic evaluation of movements: *individual features* of the observer (e.g., sensorimotor expertise and sociocultural background), and *objective features* of the stimuli (see Table 1).

**Table 1:** Studies investigating the role of objective and individual features in modulating the aesthetic evaluation of movement (dance)

Authors	Bodies	Stimulus type	Objective features	Individual features	Participants	Questions	Technique
Darda & Cross, 2021^239^	Single	Videos: Bharatanatyam and ballet dance	Dance technique	Ratings, expertise, cultural background	Experts and non-experts	Familiarity, complexity, evocativeness, abstractness, technical competency, reproducibility, beauty, liking, enjoyability	Behavioral
Christensen et al., 2021^197^	Single	Videos: ballet dance	Emotion expression, arousal	Ratings, galvanic skin response	Experts and non-experts	Emotion	Behavioral, physiological
Orlandi, Cross et al., 2020^118^	Single	Videos: contemporary dance sequences	Velocity, acceleration, smoothness, entropy	Ratings	Non-experts	Enjoyment, reproducibility, speed, effort	Behavioral, kinematic analysis
Kirsch & Cross, 2018^237^	Single	Videos: dance sequences	Performance	Ratings, multisensory training (expertise), age group	Non-experts	Likability, reproducibility	fMRI, training
Deinzer et al., 2017^240^	Single	Live dance performance	Velocity, acceleration, traveled distance	Ratings	Non-experts	Individual engagement and attention, valence, arousal, sense of body, space, and time	Behavioral
Christensen, Gomila et al., 2016^156^	Single	Videos: ballet and contemporary dance	Emotion expression	Ratings, heart rate variability, expertise	Varied expertise	Expressivity, likability	Behavioral, physiological
Christensen, Pollick et al., 2016^205^	Single	Videos: ballet dance	Motion energy, luminance, roundedness, impressiveness	Ratings	Non-experts	Valence, arousal, beauty, likability, interest	Behavioral
Kirsch et al., 2015^170^; Kirsch & Cross, 2015^238^	Single	Videos: dance sequences	Performance	Ratings, multisensory training (expertise)	Non-experts	Likability, reproducibility	fMRI, training
Kirsch et al., 2013^236^	Single	Videos: dance sequences	Performance	Ratings, multisensory training (expertise)	Non-experts	Likability, complexity, interest, enjoyability to reproduce	Behavioral, training
Orgs et al., 2013^11^	Single	Images: sequences of dance postures (apparent motion)	Symmetry, good/bad continuation	Ratings	Non-experts	Speed, aesthetic ratings	Behavioral
Torrents et al., 2013^3^	Single	Videos: animations from contemporary dancers	Different features for each of the four skills depicted	Ratings	Non-experts	Beauty	Behavioral, kinematic analysis
Neave et al., 2011^241^	Single	Videos: 3D animations from non-dancers’ kinematics	Amplitude, variability, speed	Ratings	Non-experts	Dance quality	Behavioral, kinematic analysis
Cross et al., 2011^9^	Single	Videos: classic and contemporary dance	Motion energy	Ratings	Non-experts	Likability, reproducibility	fMRI
Calvo-Merino, Urgesi et al., 2010^8^	Single	Images: ballet and hybrid postures	–	Ratings	Non-experts	Preference between two images, likability	TMS
Calvo-Merino et al., 2008^7^	Single	Videos: ballet and Capoeira	Speed, displacement, body parts, direction	Ratings	Non-experts	Simple–complex, dull–interesting, tense–relaxed, weak–powerful, like–dislike	fMRI
Sawada et al., 2003^242^	Single	Videos: modern dance	Emotion expression	Ratings	Non-experts	Emotion	Behavioral
Cross et al., 2024^243^	Dyad	Videos: contemporary and street dance	Synchrony, positioning	Ratings	Non-experts	Enjoyment, togetherness	Behavioral, fMRI
Moffat & Cross, 2024^244^	Dyad	Videos: point-light animations	Synchrony, similarity, predictability	Ratings, individual traits	Non-experts	Enjoyment, synchrony, recognition	Behavioral, fNIRS
Moffat & Cross, 2024^198^	Dyad	Videos: point-light animations	Synchrony, similarity, predictability	Ratings, individual traits	Non-experts	Enjoyment, reproducibility, synchrony	Behavioral, kinematic analysis
Hartmann et al., 2023^245^	Dyad	Videos: point-light animations	Postural synchrony, gestural simultaneous and sequential coupling, orientation	Ratings	Non-experts	Similarity, interactivity, leadership	Behavioral, kinematic analysis
Tang Poy & Woolhouse, 2020^154^	Dyad	Videos: hip-hop dancing pairs	Synchrony: between dancers, movement/music	Pupil dilation, ratings	Non-experts	Attractiveness, familiarity	Eye-tracker
Hartmann et al., 2019^199^	Dyad	Videos: point-light animations	Torso orientation, temporal and spatial coupling, vertical head synchrony	Ratings	Non-experts	Similarity, interactivity	Behavioral, kinematic analysis
Monroy et al., 2022^246^	Group	Video: different styles of dance	Synchrony	Cultural background, ratings	Non-experts	Evaluative value, complexity, arousal, control, diversity, happiness, familiarity	Behavioral
Howlin et al., 2020^211^	Group	Videos: contemporary dance	Visual motion, acceleration, synchrony, congruency movement/sound	Ratings, heart rate	Non-experts	Enjoyment	Behavioral, physiological
Vicary et al., 2017^2^	Group	Live dance performance	Visual motion, acceleration, synchrony	Ratings, heart rate	Non-experts	Enjoyment, togetherness	Behavioral, physiological

Examples are reported for the current review’s scope.

Among the individual features, the most studied is acquired experience—extensively discussed in the previous sections—which includes visuomotor expertise with the observed movement,^7^^,^^152^^,^^160^ theoretical familiarity with the movement vocabulary,^205^ and knowledge of choreographic structure.^11^ Recent studies also show that expertise interacts with sociocultural background in shaping aesthetic evaluation of movements.^239^ For example, Western and Indian participants preferred ballet and Bharatanatyam dance repertoire, respectively, indicating an in-group bias based on cultural affiliation.^239^ Notably, this effect was only found in Western non-experts (vs. experts) and was not modulated by expertise among Indian participants—suggesting a complex interaction between culture and training.

At the opposite end of sociocultural influences, interoceptive ability has also been found to impact the aesthetic evaluations of body and movement stimuli. A recent study^197^ revealed a preference for expressive (vs. non-expressive) dance videos, with expressivity ratings correlating negatively with autonomic responses (i.e., reduced skin conductance). This relationship was positively linked to participants’ interoceptive accuracy; the more accurate participants detected their internal body signals (i.e., higher interoceptive accuracy), the stronger the link between perceived expressivity and psychophysiological responsiveness to each clip.^197^

Beyond individual differences, substantial attention has been given to *objective features* of movement that influence aesthetic evaluation. These include, but are not limited to, symmetry,^11^ complexity,^118^ movement type (e.g., vertical jumps with horizontal displacement, fast turns), movement repertoire,^3^^,^^7^^,^^220^ temporal dynamics,^240^ emotional expression and recognition,^242^ and the connection between movements and sound.^211^^,^^212^^,^^247^ For instance, in two studies by Orgs et al., observers preferred sequences of static body postures that generated apparent motion maximizing spatial symmetry, as well as sequences that created “good” spatial continuations, compared with those characterized by path reversal.^11^ At the neural level, this “good continuation” effect was associated with increased activity in the EBA, motor, and supplementary motor area, and connectivity among these regions.^248^

In a foundational study by Calvo-Merino et al., non-expert observers preferred dance steps involving the whole body, such as jumps in place or with large spatial displacement—preferences associated with increased activity in the OTC and right premotor cortex.^7^ Kinematic analysis of dance movements also revealed that non-expert observers are more drawn to basic attributes of dance gestures—quantified by motion capture—such as faster turns, large movement amplitude, and longer balancing positions.^3^^,^^241^ Similarly, Deinzer et al. reported that audiences preferred faster over slower dance pieces during live performances.^240^

One central topic in aesthetics research is *stimulus complexity—*a concept widely studied in music^249^^,^^250^ and visual arts,^251^^,^^252^ but still emerging in body and movement research. Prior findings illustrate that the definition of complexity critically influences its relationship with aesthetic evaluation.^253^ A recent study investigating action timing in the aesthetic evaluation of complex dance sequences^118^ applied an information theory approach, presenting non-dancers with contemporary dance fragments performed in either a fluent, uniform style or a varied style (including accelerations, decelerations, and pauses). Complexity was quantified using motion smoothness and entropy derived from the speed and acceleration profiles, serving as measures of movement variability and predictability. Results showed that observers preferred sequences that were varied yet predictable, and rated them as faster, more effortful, and more difficult to reproduce.

Another widely studied topic in dance neuroaesthetics is the relationship between movement kinematics and whole-body emotion expression and recognition.^212^^,^^242^ This relationship has been studied across various contexts, including walking,^254^ everyday actions,^255^ dynamic arm gestures in social situations,^256^ freestyle dancing,^257^ and formal dance techniques.^205^^,^^220^ When participants are invited to freely move (dance) to music, happy expressions are characterized by faster, more accelerated, and more expanded movements, in contrast to sad expressions.^257^ Similarly, when observing professional dancers, emotional expressions are often influenced and predicted by movement acceleration,^242^ as seen in sadness (e.g., lower acceleration) and anger (e.g., higher acceleration and shorter traveled distance). Non-dancer participants also associate positive emotions with rounded (vs. edgy/non-round) body shapes and movements that require a high level of skill and greater spatial expansion (e.g., *leg lift à la seconde*), with emotional valence evaluations (but not arousal) correlating with physical parameters of the stimuli, such as motion energy and luminance.^205^

As with aesthetic evaluation, several studies suggest an interaction between individual characteristics (e.g., motor expertise) and objective stimulus features (e.g., movement kinematics) in recognizing emotions through movement. Observers’ experience, sociocultural background, and interoceptive capability likely interact with the kinematics and complexity of the stimuli, shaping both aesthetic and emotional responses. Future research should continue to investigate how these two categories of features—individual and objective—interact, with special attention to how they might be more deeply integrated in the study of movement aesthetics.

## From single-body to two-body and multiple-body interactions

### Visual processing of two-body interactions

Over the last few decades, there has been a rise in studies investigating the neurobehavioral correlates of interpersonal interaction perception, recognition, and understanding, with a focus on the role of the temporal dynamics in interaction coordination, such as rhythm and synchrony.^258^^,^^259^ A key area of research in this field focuses on spatiotemporal perceptual mechanisms (e.g., asynchrony vs. synchrony, configural vs. featural processing) and higher-order features (e.g., emotional coherence, cognitive conflict, and semantic processing) that influence how we perceive social interactions. It also explores the specific roles of visual, motor, mentalizing, and emotional systems in processing these interactions.^260^ Each of these systems may contribute to the aesthetic evaluation of interpersonal interactions.

At a behavioral level, recent studies have shown that the relative face-to-face positioning of dyad members modulates body image perception. This effect supports the idea that two-body interaction perception can be framed in terms of configural vs. featural processing. The notion that two individuals facing each other are processed configurationally, compared to those facing away, was supported by a series of studies by Papeo et al.,^261^^,^^262^ in which a significant inversion effect was observed in response to facing (vs. non-facing) body dyads—but not control objects (e.g., chairs). Specifically, the authors found reduced recognition performance for inverted vs. upright bodies when presented in a face-to-face, rather than back-to-back, arrangement. Visual search tasks also reveal an advantage in recognizing facing dyads (targets) among non-facings dyads (distractors), compared to the reverse task.^263^ According to the authors, this more efficient processing of facing dyads could be due to a rapid perceptual grouping of interacting bodies into a single attentional unit.

It is worth noting that the effect of dyad positioning on target recognition has also been observed with arrows and non-social objects (e.g., desk lamps, fans, bicycles, cars^264^^,^^265^). These findings have been interpreted as evidence of a general, not domain-specific (i.e., social interaction processing) mechanism by which constituent elements direct observers’ visuospatial attention (e.g., direction cueing account^265^^,^^266^). Further studies are needed to better understand the mechanisms underlying interpersonal interaction perception and to disentangle the contributions of low-level and high-level features to two-body visual processing.

At the neural level, studies have confirmed the sensitivity of the lateral occipital cortex (LOC) to the number and spatial relationship between bodies.^262^^,^^267^^,^^268^ Two-body visual perception engages the inferior and medial parts of the LOC more than single bodies; facing bodies engage the superior and medial portions of the LOC more than the same non-facing bodies.^268^ EBA, FBA, and FFA seem to respond more strongly to upright facing bodies than to non-facing bodies, with FFA and EBA activity additionally modulated by the orientation (upright vs. inverted) of facing vs. non-facing bodies. Notably, the EBA appears to play a crucial role in processing the relative position of bodies within dyads.^262^^,^^267^^,^^268^^,^^269^

In a recent fMRI study, participants observed point-light animations of two bodies walking toward or away from each other. These stimuli elicited increased activity in EBA and pSTS for facing (vs. non-facing) bodies, as well as in regions associated with the processing of social stimuli and social tasks (e.g., TPJ, dorsolateral PFC, IFG, precentral gyrus, and insula). Importantly, increased coupling (effective connectivity) between EBA and pSTS was observed in response to facing configurations, along with above-chance classification accuracy (MVPA) for facing bodies and single bodies within a facing configuration. As discussed by the authors, this EBA/pSTS network may act as a gateway to a larger network underlying social interactions representation,^269^ supporting the notion that social interaction perception begins as a bottom-up process in visually selective regions (for a deeper explanation, see McMahon & Isik, 2023^270^; Papeo, 2020^271^; Wurm & Caramazza, 2022^272^). A recent TMS study supports this possibility by showing that transient functional interference with left EBA activity disrupts configurational processing of facing bodies.^100^ The authors demonstrated that TMS delivered over the left EBA affects the inversion effect (i.e., reduced recognition performance for inverted vs. upright bodies) for facing—but not for non-facing—bodies. This effect was not observed with stimulation over a control region (the occipital place area).

Previous studies using various stimuli and tasks (e.g., implicit vs. explicit stimulus evaluation) indicated that, in addition to occipitotemporal regions, other brain regions contribute to social interaction processing.^273^^,^^274^^,^^275^ For instance, when participants freely explored images depicting two facing individuals (e.g., greeting by shaking hands, hugging, or touching each other on the shoulder), compared to non-facing individuals, increased activity was observed in the amygdala, dmPFC (dorsomedial prefrontal cortex), and pSTS.^275^ Additionally, the pSTS has been consistently shown to be crucial for social interaction perception.^276^ Activity in this region—and in the EBA—is modulated by the motion and interactive nature of observed interactions,^277^ as well as by different interaction scenarios (e.g., arguing, celebrating, laughing^278^; cooperativeness^279^). When participants evaluate whether two individuals are acting together or alone, observing social interactions (e.g., one actor’s action triggers the second actor’s reaction) vs. non-interactions engages areas of the mentalizing system (e.g., left TPJ, right anterior STS, dmPFC) and the AON (e.g., IFG, PM, IPS, superior parietal gyrus), in addition to the pSTS.^274^

These findings align with a recent meta-analysis by Arioli and Canessa, which suggests the existence of a “social interaction network” including key regions of the AON and mentalizing system,^273^ the latter supporting the understanding of others’ intentions, social beliefs, and personality traits.^280^ These results support a hierarchical model of interaction processing in the lateral posterior temporal cortices, progressing from visuomotor processing of individual and shared intentions to complex inferences about others’ mental states. Neural activity is proposed to propagate from the bilateral inferior–middle temporal cortex and right pSTS to bilateral pSTS, TPJ, and ultimately the medial prefrontal cortex, possibly in conjunction with the amygdala.

It is noteworthy that a previous study reported an increased recruitment of the EBA, FBA, FFA, and pSTS when observing incongruent dyadic interactions, as compared to both congruent interactions and non-interactions.^281^ The authors proposed that this increase in the extrastriate visual activity may reflect the greater processing demands required to form a coherent person percept during ambiguous dyadic interactions. In the same study, MVPA revealed above-chance classification accuracy for distinguishing between congruent and incongruent interactions in the left EBA and right FBA, with posterior insular activity parametrically modulated by perceived meaningfulness. Consistently, observing point-light animations depicting two individuals acting in a socially atypical manner (incongruent with social conventions) compared to typical actions was associated with greater activity in the bilateral precuneus and several frontoparietal and occipital regions.^282^

On one hand, this evidence suggests a role of social interaction semantics, typicality, and congruency in modulating interaction processing, which warrants further investigation. On the other hand, increased neural activity in response to incongruent interpersonal interactions is compatible with a predictive coding framework and the generation of prediction errors.^283^ Prior expectations and social knowledge may shape the activity of multisensory regions involved in understanding others’ intentions (e.g., mirror neurons^101^ and Theory of Mind^284^). Differences between (top-down signals) and sensory information (bottom-up inputs) that generate prediction errors may occur at various levels of the hierarchical processing, including the visual processing of interpersonal interactions. These differences could involve perceptual processing of low-level features of bodies (e.g., symmetry) and movements (e.g., synchrony), as well as processing of high-level contents by cognitive systems representing interactions meaning, emotional valence, or social norm compliance.

Besides relative positioning (e.g., face-to-face) and the semantic/emotional meaning of body postures, another crucial feature in the neural processing and evaluation of interpersonal interactions is synchrony. This applies to both two-body and multiple-body/group-level interactions. Sensitivity to interpersonal synchrony has been observed in dance postures, where synchronous movements may be more easily perceived as forming a coherent whole compared to asynchronous or asymmetric movements. This hypothesis is supported by an EEG study using a frequency tagging procedure (i.e., stimuli presented at different flickering rates to generate Steady-State Visual Evoked Potentials), which reported stronger responses for synchronous, fluent single-body movements that appeared bound together.^285^ Additionally, in free-dancing dyads, perceived interactivity and similarity between two bodies were linked to movement similarity based on periodicity (i.e., periodic locking).^286^

Altogether, specific features within a dyad or group (e.g., relative positioning^261^ and synchrony)^285^ can seem to facilitate configurational processing. The question of which combination of body features—visuospatial, temporal, spatiotemporal—support this processing has only recently begun to be explored. Despite growing interest in interpersonal interaction perception and understanding, the aesthetic evaluation of two-body interactions and groups remains largely unexplored. While possible reasons for this lack of evidence will be discussed later, the few behavioral and neuroimaging studies that have investigated the visual aesthetics of people moving together will be covered in the following paragraph.

### Action timing in group aesthetics

Recently, research on movement aesthetics has expanded beyond the single-individual approach to include the aesthetic evaluation of two-body interactions and groups of individuals moving together. These studies have focused on action timing, in particular, on how movement synchrony among performers impacts the aesthetic response of observers.^2^^,^^154^^,^^198^^,^^203^^,^^243^^,^^246^^,^^285^

In a study by Vicary et al.,^2^ a group of non-dancers performed a choreography involving arm-swinging, walking, and running, designed to modulate the level of synchronization among performers. Inertial sensors recorded the dancers’ upper limb acceleration, while observers’ heart rates were tracked using wrist sensors during live performances. Observers continuously rated the performance for enjoyability and togetherness. Overall, dancers’ timing dynamics predicted aesthetic and affective (arousal) responses in observers, suggesting that performer coordination influences audience aesthetic evaluations. In a separate study, viewing synchronous (vs. asynchronous) movement among hip-hop dancers was linked to higher attractiveness judgments and pupil dilation, possibly indicating greater salience for synchronous interpersonal movements.^154^

Recently, Moffat and Cross had non-dancer participants evaluate the synchrony level, reproducibility, and enjoyability of videos depicting pairs of stick figures moving in low- or high-synchrony.^198^ Results indicated an underestimation of actual synchrony. Additionally, synchrony evaluation accuracy was positively associated with enjoyment and reproducibility ratings for high-synchrony movements, and with computed predictability and body competence for low-synchrony movements. Enjoyment was also positively related to computed similarity, perceived reproducibility, and observers’ empathy traits, while low enjoyment ratings correlated with greater awareness of bodily signals.

Monroy et al.^246^ found that observers’ cultural background (Western vs. Eastern) mediated the relationship between performers’ synchrony and aesthetic responses. Although all participants rated synchronous (vs. asynchronous) group dance videos as more uniform, calm, familiar, and happy, cultural background influenced their aesthetic evaluations of synchronous vs. asynchronous group movements.

From the performers’ perspective, evaluations of group affiliation, member likability, and conformity (i.e., prosocial behavior) seem linked to engagement in coordinated movement sequences (distributed coordination), rather than strict group synchrony.^287^ Moreover, individuals’ enjoyment of moving together increases as their action timing aligns more closely with that of the group.^203^

While these studies have identified a relationship between changes in the timing of performers’ movements and audience engagement, the neural bases of this phenomenon require further investigation. In an fNIRS study by Moffat and Cross,^244^ a subset of stimuli from their prior study was selected, and non-dancer participants were instructed to replicate movements performed by a stick figure. Participants then evaluated the enjoyability, synchrony, and novelty of the same or novel movements performed by pairs of stick figures. Enjoyment ratings were higher for previously performed and recognised movements, and their ratings were associated with increased left STG response. Better recognition of performed movements was associated with increased activation in bilateral STG, left IFG, and right IPL.

While this study suggests a role of regions considered part of the AON for the enjoyment of interpersonal synchronization, there is also some evidence that brain areas beyond the AON are involved in the observation and aesthetic evaluation of dyadic dance movements. Pollick et al.^213^ investigated the collective experience of watching dance by showing participants a long video of a dance performance featuring a duo dancing to music during fMRI scanning. Afterward, participants completed a behavioral memory task, identifying whether a series of short videos were “old” (from the initial performance video) or “new”. The left precuneus showed increased activity in response to remembered (vs. forgotten) dance sequences, while the right cerebellum showed the opposite pattern. Additionally, functional connectivity analysis on brain regions activated during dance observation (using intra-subject correlation-ISC) identified eight subnetworks. The activity of six subnetworks was attributed to sensory and motor aspects of human action observation, while that of two subnetworks was associated with more complex cognitive processes, such as attention allocation and access to internal information (e.g., default mode network), suggesting that regions outside the AON may contribute to the processing of dance.

In line with this, Cross et al.^243^ recently examined how movement synchrony (vs. asynchrony) and mutual gaze (vs. facing away) influence observers’ enjoyment and togetherness ratings of observed dyadic dance movements. Observing synchronous (vs. asynchronous) movements was associated with higher ratings for both enjoyment and togetherness, while mutual gaze influenced togetherness ratings. At the neural level, engagement of brain areas within the AON and Theory of Mind networks was modulated by both synchrony and mutual gaze, with several regions showing a stronger association with togetherness ratings than with enjoyment ratings. For example, during the observation of asynchronous movement, togetherness ratings were linked to activity in the AON (bilateral dmPFC, IFG, IPL; left LOC and right STS). Additionally, when the dancers moved asynchronously but faced each other, togetherness ratings were associated with activity in the left temporal pole, a key region within the Theory of Mind network.

In conclusion, since action timing is fundamental for coordination and interaction between individuals in the social context^288^^,^^289^ and performative arts,^290^^,^^291^ it is unsurprising that most studies on moving group aesthetics have focused on such a topic. At the same time, movement types (e.g., jumps, turns) and visuospatial attributes between multiple bodies^199^^,^^245^—such as horizontal and vertical displacement—may also play a crucial role in modulating aesthetic preferences during action observation and warrant further investigation.

### Toward a neuroaesthetics of interactions

Based on the available evidence, most studies examining movement evaluation—particularly in dance—have focused primarily on evaluating stimuli that showcase individual performers.^7^^,^^9^^,^^11^^,^^118^ Only recently has attention shifted toward investigating the timing of actions in group performances.^2^^,^^203^ We propose that the aesthetic evaluation of two-body interactions, as observed in dance, may rely on the activity of perceptual, sensorimotor, emotional, and cognitive systems that underlie interpersonal processing and broader social functions. These systems may be less activated by single-body movements that lack interactive meaning or features. Just as prior studies have shown that the aesthetic evaluation of objects or non-bodily art is linked to systems engaged in sensory, perceptual, cognitive, and evaluative processing, we suggest that the aesthetic evaluation of two-body interactions may involve complex cortical and subcortical systems sensitive to the relational dynamics and emotional evaluation of bodies and their movements. These systems encompass processing of multiple bodies’ movements, their relational meaning, and affective value. Reciprocally, aesthetic evaluation of two-body interactions may enhance social functions by representing a powerful and rewarding class of stimuli that facilitate interpretation of individual body elements based on their relations. Under these assumptions, dyadic or group dance material represents a fruitful stimulus for triggering the processing of visual configurations created by different bodies and their movements, as well as the emotional and semantic meaning of their dynamic interaction.

In this regard, interaction aesthetics may be supported by an interplay between different brain networks, including: (1) occipitotemporal visual regions (e.g., EBA and STS), sensitive to two-body positioning and potentially other visuospatial features; (2) sensorimotor regions (e.g., within the AON) that may be engaged based on observer’s familiarity and expertise with the movements of one or both bodies and the specific task proposed; (3) frontal and reward-related structures linked to social cognition (e.g., OFC, amygdala, insula); (4) regions involved in higher-order social processing (e.g., Theory of Mind: TPJ, mPFC), which may play a role in evaluating dance interactions (e.g., dramaturgy). Building on evidence from Cross et al.^243^ on two-body aesthetics, along with work by Jang and Pollick^167^ on single-body movement—both suggesting an interaction between brain networks involved in sensorimotor processing of movement (i.e., the AON) and the mentalizing system (e.g., Theory of Mind)—we support the idea that, within the context of multiple-body interactions, dance could serve as a gateway to explore social cognition.

Below, we outline nine conceptual and methodological aspects that we believe warrant attention in future studies of the aesthetic evaluation of two- or multiple-body (dance) interactions. These considerations will contribute to defining the “*neuroaesthetics of interactions”*.

#### Conceptual aspects

(1) *The role of semantics in two- or multiple-body aesthetics.* In exploring how body interactions are perceived and evaluated, dance-inspired body postures and movements are valuable due to their non-symbolic nature. Unlike daily actions or sports-related movements, which often convey specific meanings (e.g., a handshake as a greeting or a high-five as celebration), dance movements allow for combinations without semantic conflicts.^281^^,^^292^ This makes it possible to investigate the aesthetic evaluation of interactions independently of semantic content, focusing instead on underexplored low-level features such as positioning and distance between bodies. However, understanding how performers and choreographers convey meaning through kinematic features and non-verbal communication (e.g., dramaturgy, semiotics^293^) requires further neuroscientific investigation. While previous studies have addressed aspects of non-verbal communication in dance,^180^^,^^183^ more systematic research is needed.
(2) *The role of social emotions and attention in two- or multiple-body aesthetics.* In single-body aesthetics, the relationship between movement features (e.g., velocity, acceleration, and roundedness) and emotion recognition is well documented,^205^^,^^242^ as discussed in section dance aesthetics: the role of individual and objective features. For instance, acceleration distinguishes positive emotions (e.g., happiness) from negative ones (e.g., sadness).^257^ Accordingly, the emotional content of interacting bodies may depend on the congruence or incongruence of their acceleration profiles. These low-level features may also guide the observer’s attention toward a particular body. Currently, the link between affective responses, attention, and aesthetic evaluations of interacting bodies remains speculative and requires empirical testing. Additionally, complex movement sequences—as in choreography^210^ or social interactions^294^—can influence engagement and interest. Since affective responses to structured movement are dynamic, they warrant more sophisticated continuous measurement approaches.
(3) *The role and new definition of configurational processing for aesthetic evaluation.* It remains unclear how configural visual processing of interactions influences perception and modifies emotional, semantic, and aesthetic evaluations. If configural processing reflects the contribution of neural systems specialized in part-whole relationships, it could significantly impact how interacting bodies are aesthetically judged. While configural processing is well established for canonical orientations of bodies and faces (e.g., upright oriented versus inverted),^47^^,^^49^ studies examining configural processing of two-body interactions are still emerging.^268^^,^^281^ Research suggests that this form of processing is more likely when bodies face each other^261^ or move in synchrony.^2^^,^^285^ Further work is needed to explore how orientation, posture, distance, gesture, and spatial symmetry shape the aesthetic experience of dyadic interactions.
(4) *The role of individual features such as demographics, personality, and sociocultural background in interaction neuroaesthetics.* The aesthetic evaluation of two-body and group interactions is likely influenced by the observer’s characteristics. As in single-body aesthetics, factors such as dispositional traits (e.g., empathy),^141^ demographics (e.g., sex),^124^^,^^126^^,^^137^ ethnicity (e.g., the other-race effect),^295^ and visuomotor familiarity or expertise with interactions (see below) may significantly shape interaction evaluation. In this context, the dichotomy between objectivist and subjectivist theories becomes limiting: while consistent movement features may lead to shared preferences (objectivist), individual traits can modulate and override these effects (subjectivist). To understand these mechanisms, studies should consider both stimulus features and observer characteristics—and do so across multiple art forms (e.g., comparing dance and visual art^239^).
(5) *The role of acquired expertise in interaction neuroaesthetics.* As demonstrated in single-body studies, expertise in observing specific movement repertoires modulates AON activity and subjective evaluations.^170^^,^^236^ With multiple individuals moving together, expertise or familiarity becomes more complex, yet remains critical. For example, if an observer is familiar with the sequence of only one dancer in a dyad, attention may be disproportionately directed to that individual. This opens new directions for investigating how movement features and prior experience shape perception and attention. Short-term training paradigms could manipulate expertise with distinct “quality of movement”—including posture, spatial arrangement, and timing—all of which are core to dance vocabulary.^200^^,^^201^

#### Methodological aspects

**Figure 2:**
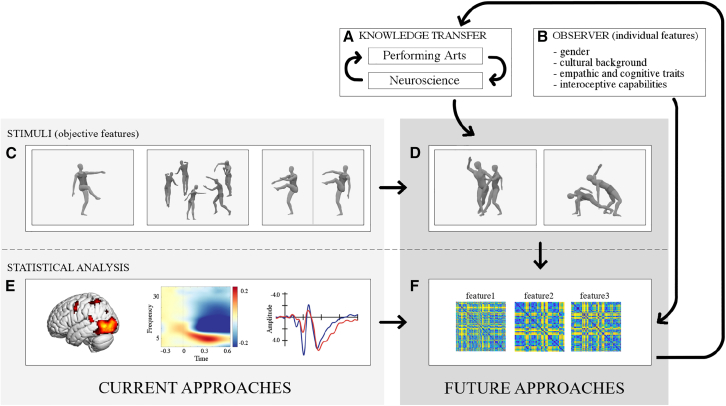
The figure illustrates current (left) and future (right) approaches to investigating two-body aesthetics The majority of studies have focused on single-body presentations, and more recently, on groups of individuals moving together (C). The two-body presentation in this context was achieved by juxtaposing two independently recorded videos. Here, we propose a more refined approach, examining real body interactions with varying visuospatial and temporal features (D). Additionally, transitioning from univariate (E) to multivariate (F) statistical methods would facilitate a better understanding of how multiple objective stimulus features interact to shape aesthetic evaluations of interactions. Furthermore, objective stimulus features (D) should be considered in combination with individual characteristics of the observers (B) to modulate interaction aesthetics. Finally, a dialogue between neuroscientific and performative research on body and movement aesthetics is strongly encouraged, fostering a full circle of knowledge transfer between those two complex frameworks (A).
(9) *Collaboration between experimental psychologists, neuroscientists, and performative professionals.* As illustrated in Figure 2, research on two-body and multiple-body interactions—particularly in dance—would benefit greatly from collaboration between neuroscientists and professionals in the performing arts (panel A). Such collaboration would enable the design of more natural, ecologically valid, and complex stimuli, including body postures and visuospatial relationships between bodies. Currently, many studies assess body interaction evaluations (e.g., interactivity, similarity, and likability)^243^^,^^246^ using juxtaposed two images or videos of separately recorded bodies, which interrupts the interaction (panel C). To study how visuospatial and temporal features modulate aesthetic evaluation, more complex and truly interactive stimuli are needed—such as entangled or visually intertwined bodies (panel D). The broader range of stimulus features in these stimuli would also require the development of automated methods to extract and quantify movement parameters (e.g., kinematic analysis). These objective features should then be analyzed in combination with observer characteristics (e.g., expertise, gender, sociocultural background) to understand how both contribute to positive aesthetic experiences (panel B). To support this complexity, multivariate statistical approaches (panel F) should be preferred over traditional univariate methods (panel E).

(6) *Defining and quantifying relevant dimensions of interactions.* As shown in studies on music and visual art, clear and meaningful definitions and quantifications of interactions’ global features are essential for investigating their emotional, semantic, and aesthetic dimensions.^252^^,^^253^^,^^296^ These definitions can focus on temporal features, visuospatial features, or their combination. For example, when temporal dynamics are the focus,^118^ an identical movement set may evoke different responses depending on whether it is performed simultaneously, sequentially, or with a time delay—regardless of spatial configuration. Visuospatial features also influence aesthetic evaluation, such as distance between bodies (e.g., near, far), positioning (e.g., face-to-face, back-to-back), posture (e.g., upper body bent), and vertical space use (e.g., standing, seated). Objective features previously linked to single-body aesthetics (e.g., implied motion, symmetry) should be assessed for each body individually and also for the dyad or group as a whole (e.g., configurational processing). For instance, in freely dancing dyads, perceived interaction has been shown to depend on torso orientation, while similarity ratings are best predicted by temporal and spatial coupling.^199^^,^^245^
(7) *The impact of ecological stimuli and context on interaction neuroaesthetics.* Another essential consideration is the ecological validity of the stimuli and the influence of the context on aesthetic experience. In neuroscientific studies, bodies are often presented without faces or heads to avoid confounds from facial emotional expressions,^36^ or to avoid gaze-related attention shifts in social cueing studies.^297^ Body postures are typically set against neutral backgrounds to isolate specific bodily and kinematic features, excluding original contextual information.^298^ Point-light animations offer a simplified, extreme example.^56^ These techniques help separate configurational and analytical processing of body form and motion, but they also distance perception from real-life experience. Social interaction perception and movement aesthetics are complex phenomena, shaped by contextual information.^69^^,^^273^ In dance especially, effective communication and narrative construction^293^ often rely on additional elements—such as music, costumes, and scenography.^293^ To capture the richness of real-life interaction, future research should strive to incorporate more naturalistic, ecologically valid stimuli reflecting shared space and dynamic social contexts.
(8) *Multivariate approaches to analysing stimulus features in interaction neuroaesthetics.* Interaction neuroaesthetics would benefit from a shift from univariate toward multivariate analysis approaches (see Figure 2). This would allow researchers to investigate how multiple objective features—visuospatial configurations or time dynamics of individual and group movement—interact in shaping aesthetic evaluations. In visual art research, techniques like Representational Similarity Analysis (RSA) have been used to model the complex structure of aesthetic evaluation.^299^ Recent studies on emotional body language in static and dynamic stimuli have applied RSA to identify features driving emotion recognition.^300^^,^^301^

## Discussion

As an emerging field, the neuroaesthetics of interactions presents an exciting opportunity to explore how we perceive and aesthetically evaluate interpersonal encounters. It builds on foundational knowledge and methods from various disciplines, including empirical aesthetics, performing arts, cognitive and affective neuroscience, and social cognition. At the same time, it faces important challenges, such as identifying specific perspectives for the field (6.1), addressing methodological and technical issues (6.2), and developing, updating, and integrating theoretical frameworks (6.3). In the following sections, we address these critical issues and highlight the mutual benefits both for and from other fields within and beyond neuroaesthetics.

### Three emerging perspectives on the neuroaesthetics of interactions

The first consideration involves the need to deepen topics discussed in the previous sections, along with their associated neurocognitive substrates. Future studies should investigate how attention, emotional, and semantic evaluations relate to the aesthetic evaluation of interactions, taking into account stimulus features (e.g., complexity, ecological validity), individual characteristics (e.g., expertise and sociocultural background), and active interpretation. These topics can be grouped into three emerging themes in the neuroaesthetics of interactions: (1) understanding the specific *perceptual processing* underpinning interaction neuroaesthetics and its link to meaningful stimulus properties; (2) understanding the role of *individual differences* in the aesthetic evaluation of interactions; and (3) framing interaction neuroaesthetics within a *predictive coding framework*.

(1) The first theme focuses on *perceptual processing* and the need to identify “perceptual rules” underlying aesthetic evaluation of two-body and multiple-body interactions. This may involve classical approaches used in single-body aesthetics—such as high- and low-spatial stimulus filtering or inverting the stimulus orientation—as well as new manipulations targeting specific interaction features. While many objective stimulus features have been examined in single-body perception, their applicability to dyads or groups requires further investigation. Key areas include preferences for certain movements (e.g., jumps and fast turns^7^), the role of symmetry in postures and movement sequences,^11^ temporal features such as smoothness,^118^ contextual information,^69^ and ecological validity. Particular attention should be paid to spatiotemporal features that support configurational (as opposed to local) processing of multiple static and dynamic multi-body displays. Prior research highlights the relevance of orientation and positioning,^261^^,^^286^ but a more comprehensive analysis is needed. Additionally, the impact of different definitions of stimulus complexity should be explored.
(2) The second theme centers around *individual differences* among observers that modulate interaction aesthetics, including sex,^126^ sociocultural background,^239^ personality traits,^302^^,^^303^ and perceptual or motor experience with observed movements.^170^^,^^238^ For example, cross-cultural approaches could reveal variations in neurocognitive processes related to social cognition skills, shaped by education, language, and behavior.^304^ Understanding how these factors affect aesthetic responses—such as interpersonal distance preferences^305^—could reveal universal and culturally specific aspects of social cognition. Additionally, exploring how these factors interact remains critical. While their influence on art^145^^,^^306^ and music perception^307^^,^^308^ has been documented, their role in body, movement, and dance aesthetics requires further investigation
(3) The third theme frames aesthetic evaluation of interactions in terms of predictions based on prior expectations and their violations (i.e., *predictive coding*),^101^ where the aesthetic evaluation results from minimizing prediction error across levels of the cortical hierarchy involved in movement representation (intentions, goals, motor programs, kinematics). This predictive coding approach aligns with findings on single-body movement^309^ and dance perception^103^ and has been proposed as a framework for social cognition broadly.^283^ For instance, Cross et al. found increased AON activity during observation of rigid, robotic-like movements versus natural human movements, suggesting greater top-down modulation or prediction error.^103^ The authors proposed that the mismatch between unfamiliar or unpredictable movements and expectations grounded in the observer’s own action system generates prediction error, leading to greater AON engagement. Similarly, when observing interacting bodies,^281^ we might hypothesize that expectation violations influence aesthetic, emotional, and semantic evaluations at different levels. These levels may involve physical features of the stimuli (e.g., static and dynamic features), as well as higher-level cognitive dimensions, such as emotional or semantic congruency. This interplay between aesthetics and prediction error would align with similar frameworks proposed for music^310^^,^^311^ and visual art^312^^,^^313^ perception and evaluation.

### Open challenges, methodological and technical issues for the neuroaesthetics of interactions

Another critical aspect of advancing the neuroaesthetics of interactions involves the technical and methodological developments required to fully exploit the potential of this interdisciplinary field. These developments include, but are not limited to, methods for estimating and quantifying the spatial, postural, and kinematic features of complex dyadic stimuli, as well as improving techniques to measure physiological and neural responses during live performance observation and group observation.

(1) Moving beyond the classical stimulus manipulations commonly used in social cognition studies, such as vertical and horizontal flipping bodies within a dyad, it is clear that more refined methods are necessary to capture the increased visuospatial complexity between bodies. Panel D of Figure 2 provides examples of intricately intersected body dyads that cannot be easily categorized as simply facing or non-facing; these configurations may involve varying levels of vertical space and other features, such as symmetry, distance between bodies and heads, and overlap between body parts. The use of motion-tracking systems in stimulus creation enables more accurate quantification of body features (e.g., calculating the angle between bodies) and can support the creation of 3D avatars for more controlled manipulation of stimuli in experimental settings. Alternatively, offline kinematic analysis with computer vision algorithms allows for extraction of similar body features for more complex analyses, such as quantifying peripersonal and interpersonal space around the bodies. These methods, already employed in single-body perception studies and dyadic interaction tasks in social cognition^288^ and music and dance research,^314^ would be valuable if extended to aesthetic research.
(2) The development of virtual reality (VR) environments and wearable headsets offers a promising avenue for advancing our understanding of cognitive dynamics in interaction aesthetics. VR systems are already widely used in fields such as social neuroscience^315^ and empirical aesthetics, and in rehabilitation settings.^316^ Their application in interaction aesthetic research could allow for manipulation of the observer’s perspective (e.g., first-vs. third-person) to investigate how perceived embodiment and agency influence interaction aesthetics. Additionally, VR enables modulation of the level of detail (ecological validity) in the bodies and backgrounds of observed interactions, as is currently practiced in social neuroscience. Through 3D graphic environments, stimuli can be dynamically adjusted based on real-time participant responses, including behavioral, physiological, and neural data (e.g., neurobiofeedback), facilitating studies on how body-brain interactions shape aesthetic evaluations.
(3) Another methodological challenge in the neuroaesthetics of interactions is integrating various wearable devices that track body and eye movements, physiological signals (e.g., heart rate, skin conductance), and neural activity (via EEG and fNIRS) of individuals observing (dance) interactions.^12^ The neuroaesthetics of interactions represents a testing ground for wearable and portable physiological technology for the integration of behavioral, autonomic, and neural measures—essential for disentangling the complex interplay of attentional mechanisms, emotional responses, and subjective evaluations in social and dance interactions.

### Translational perspectives of the neuroaesthetics of interactions

The third aspect concerns the opportunities and potential to translate knowledge from the neuroaesthetics of interactions into practical applications.

(1) As demonstrated, visual and sensorimotor regions, along with systems linked to reward and social processing, are engaged during the observation of bodily stimuli and play a role in their aesthetic evaluation. These areas are likely to play a similarly crucial role in evaluating the aesthetic attributes of social interactions. Understanding how specific bodily and movement attributes in dyads or groups are perceived and evaluated aesthetically could enhance social cognition abilities such as interpreting interactions and attributing value to them. This knowledge could also support the creation of complex dyadic stimuli and the quantification of specific features linked to visual configurational processing in body dyads.
(2) The neuroaesthetics of interactions offers potential for designing stimuli that engage the reward system effectively. Given that this system responds to both social engagement^317^^,^^318^ and the aesthetic pleasure associated with the observed movement,^167^^,^^170^ insights from this field could reveal how it shapes our experience of observed interactions—from goal-directed joint actions to more abstract, non-symbolic dance.
(3) Another area for mutual enrichment between fields is the application of methods and paradigms for studying human interactions to the investigation of human-robot interactions (HRI), and vice versa. There is a growing interest in exploring how interactions with humans differ from those with non-human partners, such as virtual avatars and robots.^319^ The neuroaesthetics of interactions is well positioned to contribute to social robotics and robot engineering, covering a diverse range of human-non-human encounters. For instance, studying how movement timing between humans and robots (e.g., synchrony, fluency) modulates aesthetic and emotional responses could inform the design of social robots to be perceived as more trustworthy, friendly, and approachable.^320^ Additionally, the use of artificial intelligence in dance aesthetics and choreography has recently emerged, paving the way for groundbreaking innovations.^196^
(4) Empirical aesthetics is also exploring art evaluation in settings where art naturally occurs, such as museums^321^ and theaters.^157^^,^^322^ This approach could inform performative art by providing insights into how the co-presence of multiple observers and the multisensory elements of performances enhances their impact.
(5) Following the principle of bidirectional knowledge transfer, performing arts could also draw inspiration from neuroscientific findings to tailor performances and training.^223^ For instance, motor imagery strategies, a common practice in elite sports training,^323^ are not yet fully integrated into routine dancer training. These strategies are typically used in the creative process to generate new movements, while dancers often use “marking” to refer to the practice of performing simplified versions of dance sequences during rehearsal.^324^ Similarly, insights from neuroscience on social encounters could be used to design movement tasks as starting points for artistic exploration.
(6) Understanding preferred and more enjoyable interactions between individuals can have valuable implications in clinical frameworks, particularly in therapeutic practices for individuals with social difficulties (e.g., autism and social anxiety).^325^^,^^326^ In dance movement therapy (DMT), the interpersonal dimension between therapist and client—especially through movement tasks such as mirroring^327^—is essential. Techniques used to study social cognition and interaction evaluation could be applied in clinical settings to assess the effectiveness of DMT interventions through pre- and post-testing.^327^^,^^328^

### Future directions and dance framework

This review proposes that dance offers an exceptional framework for investigating visual and sensorimotor contributions to the aesthetic evaluation of both simple movements^9^ and ecologically complex sequences.^118^ Surprisingly, there is a notable gap in studies on the aesthetics of interactive, two-body dynamics in dance, despite the inherently interactive nature of this art form, exemplified in pas de deux and ensemble performances. Dance styles such as tango, ballroom, salsa, and ballet require a high degree of coordination between partners, often evoking themes of close interpersonal interaction.^162^

Contemporary dance’s capacity to represent novel, non-symbolic postures with highly varied visuospatial characteristics enables a unique exploration of interactions between two bodies, free from confounds like incongruence effects or stereotyped emotional cues. This setting allows researchers to examine two-body features—independently or in combination—focusing on visuospatial attributes such as orientation, positioning, distance, and shared space. Moreover, the concept of interaction in dance may differ from everyday social interaction. In neuroscientific studies, two individuals typically interact in a face-to-face arrangement, facilitating social processes like shared attention.^271^ However, in dance, performers can engage in varied forms of interaction, such as: (1) mirroring the same movement from a distance without a face-to-face configuration; (2) performing the same movement with different timing (e.g., time delay); and (3) enacting different movements with shared intention and temporal/spatial linkage. For example, two dancers performing distinct movements (e.g., one opening arms horizontally, the other bending sideways) may be perceived as interacting if their movements display temporal coordination. This diversity of interactions in dance and daily life suggests variations in how individuals communicate and achieve shared motor goals, opening numerous research questions.

Given the multisensory nature of body representations in the brain^329^^,^^330^—including those related to the space surrounding the body (e.g., peripersonal space)^331^—it is plausible that somatosensory representations and associated bodily sensations may be activated during the perception of interacting individuals.^332^ These spatial representations may complement the integration of visual, somatosensory, and reward systems involved in single-body aesthetic experience.^1^

Fostering collaboration between neuroscientific researchers and experts in the performing arts, such as choreographers, dancers, and performers, is essential.^153^^,^^333^^,^^334^ Often, artists are asked to replicate actions, gestures, or sequences conceived from a scientific perspective, utilizing their expertise but not necessarily enhancing it. Bridging these interdisciplinary perspectives could lead to the creation of more ecologically valid stimuli^118^ while developing tasks and tools that also benefit artistic practice.^335^^,^^336^ An example of this synergy is seen in dance movement libraries, where professional performers contribute to designing affective gestures with both expertise and artistic sensitivity.^194^^,^^220^

Future research should increasingly consider cross-cultural and interdisciplinary aspects of dance and movement aesthetic evaluation. Currently, most neuroscientific studies on dance perception are based on Western dance techniques (e.g., ballet and contemporary dance) and target Western audiences.^118^^,^^152^^,^^197^^,^^337^ Recently, the renewed interest in dance within empirical aesthetics has expanded to include cultural traditions such as Indian (Bharatanatyam)^239^ and Iranian^338^ classical dance and comparisons between Western and Eastern observers.^246^ Steps in this direction are vital for investigating how sociocultural backgrounds modulate behavioral and neural responses to low-level kinematic features (e.g., symmetry, synchrony) and high-level interpretations (e.g., emotion perception, dramaturgy, communication) in dance.

## Conclusions

More than two decades after identifying body-sensitive regions in the OTC,^14^^,^^15^ research into the neural mechanisms underlying the aesthetic evaluation of the human body remains an active and intriguing area across various domains. While much previous research has focused on subjective and objective body features associated with single-body movement aesthetics,^7^^,^^9^^,^^11^^,^^118^ we believe that recent studies examining the time dynamics of multiple individuals moving together^2^ have catalyzed a new field: *the neuroaesthetics of interactions*.

This burgeoning field addresses the aesthetic evaluation of two-body (and multiple-body) interactions, presenting unique challenges in disentangling the various factors linked to interaction aesthetics. These complexities may have contributed to the relative lack of research to date. However, we see an exciting opportunity to advance this field by combining contemporary dance (or performing arts) and neuroscientific methodologies.^202^ This approach enables the investigation of aesthetic experiences tied to social interactions while minimizing confounding effects from familiar body postures and movements (e.g., semantic and emotional incongruence), without limiting the repertoire of available two-body postures. By merging insights from performing arts, empirical aesthetics, and cognitive neuroscience, we can leverage the strengths of each discipline to foster translational applications.

## Acknowledgments

This project has received funding from the European Union’s 10.13039/501100007601Horizon 2020 research and innovation program under the Marie Sklodowska-Curie grant agreement No 101031774. A.O. was funded by the 10.13039/501100005032Bial Foundation Grant for Scientific Research 2020/2021 (grant number: 276/20). M.C. was funded by the Sapienza University of Rome (RG120172B8343252), RG123188B4631694, RM1221816C827130, and MA22117A8A97CD42.

## Author contributions

A.O. and M.C. contributed to conceptualization, manuscript preparation, review and editing, and funding acquisition. All authors have read and agreed to the published version of the manuscript.

## Declaration of interests

The authors declare no competing interests.
